# Influenza A Pandemic (H1N1) 2009 Virus Infection in Domestic Cat

**DOI:** 10.3201/eid1603.091737

**Published:** 2010-03

**Authors:** Brett A. Sponseller, Erin Strait, Albert Jergens, Jessie Trujillo, Karen Harmon, Leo Koster, Melinda Jenkins-Moore, Mary Killian, Sabrina Swenson, Holly Bender, Ken Waller, Kristina Miles, Tracy Pearce, Kyoung-Jin Yoon, Peter Nara

**Affiliations:** Iowa State University, Ames, Iowa, USA (B. A. Sponseller, E. Strait, A. Jergens, J. Trujillo, K. Harmon, H. Bender, K. Waller, K. Miles, T. Pearce, K.-Y. Yoon, P. Nara); US Department of Agriculture National Veterinary Services Laboratories, Ames (L. Koster, M. Jenkins-Moore, M. Killian, S. Swenson)

**Keywords:** pandemic (H1N1) 2009, cat, zoonoses, case report, real-time PCR, viruses, influenza, expedited, dispatch

## Abstract

Influenza A pandemic (H1N1) 2009 virus continues to rapidly spread worldwide. In 2009, pandemic (H1N1) 2009 infection in a domestic cat from Iowa was diagnosed by a novel PCR assay that distinguishes between Eurasian and North American pandemic (H1N1) 2009 virus matrix genes. Human-to-cat transmission is presumed.

Influenza viruses are typically host specific; aquatic birds are considered the primary reservoir. However, interspecies transmission does occur (*1*–*9*) and occasionally leads to novel host-adapted strains. Interspecies transmission of influenza virus has been a public health concern because of the possibility that, through reassortment, a novel strain with zoonotic potential could emerge. The recent infection of dogs with equine influenza virus (H3N8) (*2*) and of swine with human influenza virus (H1N2) (*4*) are particularly intriguing because the former resulted in influenza becoming endemic in dogs and the latter resulted in a documented reassortment event between human and swine influenza viruses. Such concern has escalated with the recent emergence of the novel quadruple-reassorted influenza virus (H1N1) [pandemic (H1N1) 2009] in humans (*10*). Although infection and transmission of the virus have occurred primarily among humans, occasional transmission from infected persons to susceptible animals (e.g., swine, turkeys, ferrets) has been documented (*11*). The likelihood of pandemic (H1N1) 2009 infection of domestic pets has been considered less likely (www.cdc.gov/h1n1flu/qa.htm,www.avma.org/public_health/influenza/new_virus/default.asp,www.usda.gov/wps/portal/?navid=USDA_H1N1); however, we report a confirmed case of pandemic (H1N1) 2009 virus infection in a domestic cat that had been in contact with persons who had recently experienced influenza-like illness.

## The Case

A 13-year-old, castrated male**,** domestic cat that lived indoors in a single-cat household was brought to the Iowa State University Lloyd Veterinary Medical Center because of depression, inappetance, and respiratory signs of 4 days’ duration. The cat was gregarious and interacted closely with family members in the household. The family members noted that the cat was reluctant to lie in lateral recumbency and instead rested in sternal recumbency with neck extended, which was indicative of dyspnea. The cat’s vaccination status was up to date. Before the onset of clinical signs in the cat, 2 of the 3 family members had experienced an undiagnosed influenza-like illness—an upper respiratory tract infection characterized by fever, coughing, and myalgia—that lasted 3 days. Onset of the cat’s clinical signs was noted 6 and 4 days after onset of illness for the first and second family members, respectively.

At the time of examination, the cat had bilateral adventitial lung sounds (wheezes), was afebrile, and was clinically dehydrated. Radiographs of the thorax showed a bilateral caudodorsal alveolar pattern (Figure). Cytologic and microbiologic examination of bronchoalveolar lavage (BAL) fluid showed foamy macrophages (65%), nondegenerate neutrophils (25%), and small lymphocytes (10%). Clinicopathologic findings suggested a moderate, predominantly macrophagic, mixed inflammatory process. Standard microbial culture of BAL aliquots yielded no substantial growth of aerobic or anaerobic bacteria. Radiographic and cytologic findings were inconsistent with bacterial or parasitic pneumonia and not supportive of allergic airway disease. A viral cause was considered most likely; however, the cat was given amoxicillin with clavulanate (125 mg orally 2×/day) to reduce the possibility of secondary bacterial pneumonia. Notable findings from laboratory testing (complete blood count, serum biochemistry, urinalysis, and total thyroxine measurement) were moderate leukopenia characterized by a moderate lymphopenia, modest hemoconcentration, and a slightly elevated thyroxine level. Lymphopenia was consistent with acute viral infection.

**Figure:**
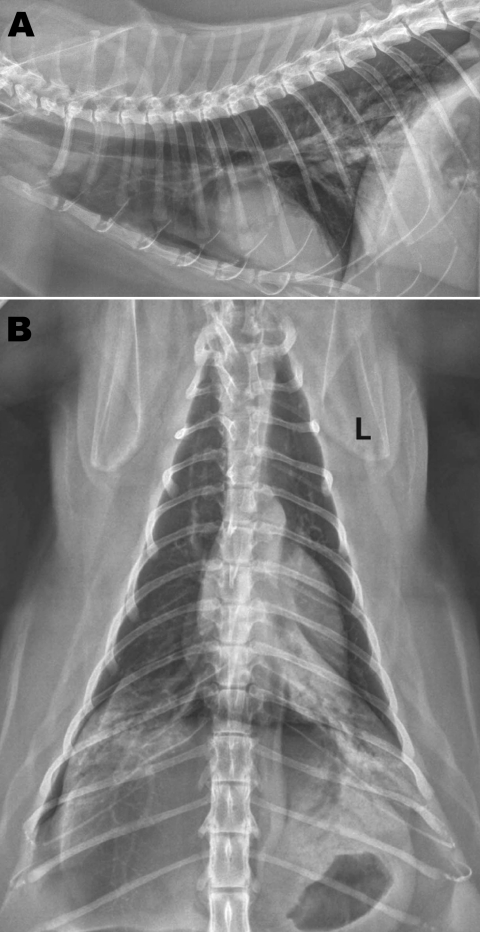
Radiographs of the thorax of a cat with confirmed influenza A pandemic (H1N1) 2009 virus infection. A) Right lateral view; B) dorsoventral view. Asymmetric soft tissue opacities are evident in the right and left caudal lung lobes. An alveolar pattern, composed of air bronchograms with border-effaced (indistinct) adjacent pulmonary vessels, is most pronounced in the left caudal lobe. A small gas lucency in the pleural space appears in the right caudal and dorsal thoracic cavity. An endotracheal tube is visible at the thoracic inlet on the lateral view in this moderately obese cat. L, left.

PCR testing (Feline URD Panel; Idexx Laboratories, Westbrook, ME, USA) of a BAL sample showed negative results for *Chlamydophila felis,* feline calicivirus, feline herpesvirus-1, *Bordetella bronchiseptica,* and *Mycoplasma felis*. Results of feline immunodeficiency virus (antibody) and feline leukopenia virus (antigen) testing (Idexx SNAP FIV/FeLV Combo Test; Idexx Laboratories) were also negative, ruling out the potential that viral-induced immunosuppression was a concurrent factor. For the following reasons we included pandemic (H1N1) 2009 on our list of differential diagnoses: recent history of respiratory disease in household family members, known widespread community prevalence of pandemic (H1N1) 2009 influenza in humans, paucity of common viral infections causing infectious caudodorsal alveolar pneumonia in adult cats, and documented susceptibility of felids to avian influenza (H5N1) (*12*,*13*). We therefore submitted a BAL sample to the Iowa State University Veterinary Diagnostic Laboratory for molecular screening and typing for influenza A and the pandemic (H1N1) 2009 virus.

RNA was obtained from the BAL fluid by using the MagMAX Viral RNA Isolation Kit (Applied Biosystems, Austin, TX, USA) and a semiautomated magnetic particle processor (Kingfisher 96; Thermo Electron Corp., Woodstock, GA, USA) according to manufacturer’s recommendations. Molecular testing used a real-time reverse transcription–PCR (rRT-PCR) influenza A screening assay specific for the nucleoprotein gene. Preliminary differentiation of pandemic (H1N1) 2009 virus from other H1 or H3 types of influenza A was performed by using an in-house rRT-PCR assay that distinguishes between pandemic (H1N1) 2009 [Eurasian matrix (*10*)] and endemic (to North America) swine H1N1 influenza viruses (North American matrix). Sequences of primers and probes are summarized in Table 1. PCRs were conducted by using the AgPath-ID Multiplex One-Step RT-PCR Kit (Ambion/Applied Biosystems) according to manufacturer’s recommendations; 10 units of Multiscribe Reverse Transcriptase (Applied Biosystems) were added per reaction. Thermocycling was performed by using the Applied Biosystems 7500 Fast Real-Time PCR System according to manufacturer’s recommendations.

**Table 1:** Oligonucleotide sequences for primers and probes and dye labels used in novel molecular testing for pandemic (H1N1) 2009 virus, Iowa State University Veterinary Diagnostic Laboratory, Ames, Iowa, USA, 2009*

Name	Sequence (5′ → 3′)	Description
Influenza A NP screening assay		
SIVRTF	CGGACGAAAAGGCAACGA	NP forward primer
SIVRTR	CTGCATTGTCTCCGAAGAAATAAG	NP reverse primer
SIVRTP	CCGATCGTGCCYTC	NP probe, MGB FAM
Pandemic influenza M differentiation assay		
M_F	TCAGGCCCCCTCAAAGC	M forward primer
M_R1	CATTCCATGAGAGCCTCAAGATC	M reverse primer 1
M_R1a	CACTCCATGAGAGCCTCAAGATC	M reverse primer 2
M_R1b	CATTCCATGAGTGCCTCAAGATC	M reverse primer 3
M_EUPr	CAGAGACTGGAAAGTGT	EU M MGB, VIC
M_NAPr	CAGAGACTYGAAGAYGT	NA M MGB, FAM

*Primers and MGB probes were obtained from Integrated DNA Technologies (Coralville, IA, USA) and Applied Biosystems Inc. (Foster City, CA, USA), respectively. SIV, swine influenza virus; NP, nucleoprotein; M, matrix.

PCR testing showed the BAL sample to be positive for influenza A virus (nucleoprotein gene), and the virus was determined to contain the matrix (M) gene of the pandemic (H1N1) 2009 virus strain. A BAL sample was submitted to the US Department of Agriculture National Veterinary Services Laboratories (Ames, IA, USA) for confirmatory testing. rRT-PCR confirmed that the BAL sample was positive for the M gene of influenza A virus and the neuraminidase (N) gene of pandemic (H1N1) 2009 virus. Sequences of primers and probes are summarized in Table 2. A cytolytic virus was isolated by using MDCK cells (*8*) and was designated as A/feline/IA/NVSL026991/2009. PCR testing of the isolate for influenza A virus (M gene) and N1 gene of pandemic (H1N1) 2009 showed positive results. Sequence analyses for hemagglutinin (HA), N, and M genes confirmed that the virus was pandemic (H1N1) 2009 virus (GenBank accession nos. GU332630 (for HA), GU332632 (for NA), and GU332631 (for M). Nucleotide homologies with the first US human pandemic (H1N1) 2009 isolate (A/CA/04/2009) were 99.4%, 99.4%, and 99.8% for the HA, NA, and M genes, respectively.

**Table 2:** Oligonucleotide sequences for primers and probes and dye labels used in confirmatory molecular testing for pandemic (H1N1) 2009 virus, National Veterinary Services Laboratories, Ames, Iowa, USA, 2009*

Name	Sequence (5′ → 3′)	Description
Influenza A M screening assay		
M+25	AGATGAGTCTTCTAACCGAGGTCG	AI M forward primer
M-124	TGCAAAAACATCTTCAAGTCTCTG	AI M reverse primer
M-124siv	TGCAAAGACACTTTCCAGTCTCTG	H1N1 M reverse primer

M+64	TCAGGCCCCCTCAAAGCCGA	M probe, BHQ, FAM
Pandemic influenza N1 differentiation assay		
N1 220F	CAACACCAACTTTGCTGC	N1 forward primer
N1 330R	GGAACCGATTCTTACACTGTTGTC	N1 reverse primer
N1 232	CAGTCAGTGGTTTCCGTGAAATTAGC	N1 BHQ, FAM

*Primers and probes were obtained from Integrated DNA Technologies (Coralville, IA, USA) and Biosearch Technologies, Inc. (Novato, CA, USA), respectively. M, matrix; AI, avian influenza; N, neuraminidase.

The cat was discharged from the medical center after diagnostic testing and correction of dehydration. A veterinarian (B.A.S.) visited the home to monitor the cat’s clinical status and administer subcutaneous fluids (120–160 mL) until the cat’s appetite improved; adventitial lung sounds resolved within 3 days. Reassessment 1 week later showed marked improvement of clinical signs but only modest improvement of the lymphopenia and radiographic findings.

## Conclusions

Because the cat was from a single-animal household and remained indoors, he was presumably infected through contact with the family members. Attempts to retrospectively confirm pandemic (H1N1) 2009 infection in the family members have been unsuccessful, but additional testing of archived biologic samples is being conducted. Although more surveillance and studies are needed to determine susceptibility of companion animals to the pandemic (H1N1) 2009 virus, possible reverse zoonotic transmission (humans to animals) remains a concern. Indeed, cases in a domestic dog and other felids have been confirmed (*11*) (www.cdc.gov/h1n1flu/qa.htm,www.avma.org/public_health/influenza/new_virus/default.asp, www.usda.gov/wps/portal/?navid=USDA_H1N1). Implications of pandemic (H1N1) 2009 virus infection in companion animals are 1) apparent human-to-animal transmission; 2) broader host range for the virus; 3) potential endemic establishment of influenza in companion animals; 4) possible transmission of influenza from companion animals to other species, including humans; and 5) the need to reevaluate companion animals as potential reservoirs or intermediate hosts for reassortment of influenza virus. This case emphasizes the need for close monitoring for interspecies transmission of influenza virus and reinforces the need for collaboration among many disciplines, a cornerstone of the One Health Initiative (www.onehealthinitiative.com).
